# A meta-learning-based robust federated learning for diagnosing lung adenocarcinoma and tuberculosis granulomas

**DOI:** 10.3389/fonc.2025.1666937

**Published:** 2025-09-26

**Authors:** Yuyao Chen, Lei Liu, Bao Feng, Yehang Chen, Jun Xu, Huan Lin, Kunwei Li, Xiaodong Chen, Yuting Ke, Haoyang Zhou, Qinghui Hu, Qinggeng Jin, Wansheng Long, Qiong Li, Xiangmeng Chen

**Affiliations:** 1 Department of Medical Imaging, Nanxishan Hospital of Guangxi Zhuang Autonomous Region, Guilin, China; 2 Laboratory of Artificial Intelligence of Biomedicine, Guilin University of Aerospace Technology, Guilin, China; 3 Department of Radiology, Guangdong Provincial People’s Hospital, Guangzhou, China; 4 Department of Radiology, Fifth Affiliated Hospital of Sun Yat-sen University, Zhuhai, China; 5 Department of Radiology, Affiliated Hospital of Guangdong Medical University, Zhanjiang, China; 6 School of Electrical Engineering, Guangxi University, Nanning, China; 7 Department of Radiology, Jiangmen Central Hospital, Jiangmen, China; 8 Department of Radiology, Sun Yat-sen University Cancer Center, Guangzhou, China

**Keywords:** lung adenocarcinoma, tuberculosis granuloma, solitary pulmonary solid nodules, SPSNs, meta-learning, federated learning, CT images, personalized federated learning signatures

## Abstract

**Background:**

Differentiating between lung adenocarcinoma (LAC) and tuberculosis granuloma (TBG) of solitary pulmonary solid nodules (SPSNs) based on CT images alone is a daunting task for clinical diagnosis. Thus, it is crucial to fully utilize CT imaging data to explore effective noninvasive diagnostic methods to improve the identification of TBG and LAC.

**Purpose:**

This study aimed to leverage CT imaging datasets from multiple hospitals for the diagnosis of TBG and LAC in SPSNs. It achieved this by deploying a meta-learning method within a federated learning framework while protecting data privacy.

**Methods:**

A total of 1,026 patients, along with their CT images of solitary pulmonary solid nodules (SPSNs) and corresponding clinical data, were collected from six medical institutions. Subsequently, the data from these six institutions were systematically partitioned into five cohorts. Each cohort was divided into two parts: the training set and the test set. A meta-learning-based robust federated learning model by training set data was proposed to construct personalized federated learning signatures (PFLS) without uploading raw data from each medical institutions. Receiver operating characteristic curve (ROC), area under curve (AUC), decision curve analysis (DCA), net reclassification improvement (NRI) and integrated discrimination improvement (IDI) are used to analyze the performance of the PFLS.

**Results:**

The PFLS trained by the proposed meta-learning-based robust federated learning framework shows superior performance compared to alternative methods. The AUC range on the training sets of the five cohorts is 0.866-0.939, AUC range on the testing sets is 0.808-0.927). The significant difference of AUC between the proposed method and the clinical model was demonstrated by the NRI and IDI. The decision curves indicated a higher net benefit of our proposed method.

**Conclusion:**

The PFLS mitigates overfitting issues arising from limited sample size in local hospitals. It also alleviates the problem that a single global model is not applicable to all hospitals due to the heterogeneity of data distribution among different hospitals.

## Introduction

The prevalence of CT has been led to a significant upsurge in the detection rate of Solitary Pulmonary Solid Nodules (SPSNs) (1). Clinically, SPSNs can be bifurcated into benign and malignant categories. Lung Adenocarcinoma (LAC) is the most common pathological type of malignant SPSNs, while Tuberculosis Granulomas (TBG) is the common pathological type of benign SPSNs (2, 3). However, the treatment regimens and clinical outcomes for lung adenocarcinoma and tuberculous granulomas are entirely different. Radical surgical resection is the preferred treatment for the former, while the latter is often managed with anti-tuberculosis medications (4). Misdiagnosis can lead to uncontrollable disease progression and a poor prognosis in patients with lung adenocarcinoma. Conversely, it may also result in overtreatment for those with tuberculous granulomas (5).

Although CT scans can identify SPSNs, the differentiation between LAC and TBG based on CT images alone presents a daunting task for clinical diagnosing. This is primarily because LAC and TBG both exhibit similar lobulated and spiculated features, and there is a lack of effective contrast agents to aid in distinguishing TBG from LAC (6, 7). Most patients with SPSNs detected by CT undergo biopsy diagnosis to guide the treatment plan. However, when the lesion is small and difficult to locate, the difficulty and related risks increase significantly (8, 9). Consequently, it is crucial to fully utilize CT imaging data to explore new effective non-invasive diagnostic methods to improve TBG and LAC identification.

Deep learning, as a data-driven technology for model performance, has shown great potential in image classification. Previous studies have demonstrated that deep learning models can extract features from raw medical images at various levels of abstraction (10, 11). Applying deep learning techniques to computer-aided diagnostic systems holds promise for improving the accuracy of TBG and LAC differentiation. However, due to the need for medical data privacy protection, medical centers are generally not allowed to share data, which limits the scale of the data. Unfortunately, robust and accurate deep learning models require a large amount of data for training; otherwise, overfitting is prone to occur, leading to a decline in the generalization ability of deep learning models.

Federated learning facilitates multi-clients collaborative training by aggregating local model parameters of each client into the shared global model, without sharing data from different clients (12). This approach fully utilizes information of each hospital without sharing raw CT image data, thus addressing privacy concerns and limiting overfitting. The federated averaging algorithm of most federated learning methods weights the parameters of each local model according to the sample sizes of different medical institution (13, 14). However, Additionally, data heterogeneity caused by differences in data collection across medical institutions (such as scanning equipment, imaging parameters, population characteristics, etc.) significantly restricts the performance of federated learning models in multi-medical institution medical image analysis (15, 16). Therefore, when there are differences in the data distributions across multiple centers, it is challenging for a single global model obtained merely by aggregating the parameters of each local model to perform consistently well across all centers (17, 18).

In this paper, a meta-learning-based robust federated learning approach is proposed to leverage heterogeneous CT imaging datasets from multiple medical institutions for the diagnosis of TBG and LAC in SPSNs. The reptile algorithm of meta-learning is deployed to aggregate gradients of parameters of each local model. This improves the performance and robustness of the global model on data from each local medical institution. Finally, each center fine-tunes the global model based on local data to complete model personalization.

## Materials and methods

### Patients

This retrospective study was approved by the Institutional Review Boards of the participating hospitals, with a waiver of informed consent. Detailed inclusion and exclusion criteria are provided in **Supplementary S1**. Finally, a total of 1,026 samples from six medical institutions. Since one medical institution has only 17 cases, we merged the data of this medical institution into another hospital, so there are a total of 5 cohorts. These five cohorts include: cohort 1 (2014–2020): 270 patients (training set: 161; test set: 109), cohort 2 (2013–2016): 87 patients (training set: 51; test set: 36), cohort 3 (2014–2019): 119 patients (training set: 70; test set: 49), cohort 4 (2011–2020): 471 patients (training set: 282; test set: 189), cohort 5 (2018–2020): 79 patients (training set: 46; test set: 33).

### CT image acquisition and evaluation

Chest CT images were acquired from six different scanners (Siemens, Toshiba, GE, Philips) with patients in the supine position, covering the entire chest from the thoracic inlet to the adrenal glands during a breath-hold. Scans were performed in spiral mode with a tube voltage of 120 kVp and automatic mAs adjustment. Images were reconstructed with standard and high-resolution algorithms at 1.0–3.0 mm slice thickness and 0.8–3.0 mm interslice gap. Two independent chest radiologists, blinded to clinical information, assessed the images using lung and mediastinal window settings, evaluating nodule location, size, margin, lobulation, and spiculation; discrepancies were resolved by consensus. Detailed information is provided in **Supplementary S2**.

### Pathological diagnosis

All samples were fixed in formalin and subsequently stained with hematoxylin and eosin (HE). The experienced pathologists performed the pathological analysis of the surgical specimens in accordance with the 2011 International Association for the Study of Lung Cancer/American Thoracic Society/European Respiratory Society classification system, and the 2015 World Health Organization (WHO) classification of lung neoplasms (19, 20). These pathologists were blinded to the CT findings.

### Image preprocessing

For neural network processing, preprocessing operations are applied to the CT images. An experienced radiologist utilizes a rectangular bounding box to crop the region of interest (ROI) from each CT slice initially. All ROIs are then interpolated and standardized to 224×224 pixels. Next, the ROIs from three sequential single-channel CT slices for the same patient are merged to form a three-channel image with the dimensions 224×224×3. Finally, these three-channel images are used as input data for the neural network. Detailed information is provided in **Supplementary Figure S1**.

### Building the meta-learning-based personalized federated learning signature

In order to adapt to the data situation of each medical institution, we train a personalized federated learning signature for each medical institution. This usually involves three steps: feature extraction, feature selection, and classifier training.

During the feature extraction process, a federated learning based on model agnostic meta-learning is used to extract the CT features of each hospital. The entire training process of federated learning encompassed three stages.

FedAvg stage: In the initial iteration of the FedAvg stage, both local and global models start with identical parameters pre-trained on ImageNet. Each local client trains its model using its own dataset. After all local clients complete training, they upload their model gradients to the global server. The global server aggregates these gradients by weighting them according to each client’s sample size relative to the total samples across all clients. The global model is then updated using these weighted gradients and distributed back to the local clients as the initial parameters for the next iteration. This process repeats for several iterations, and the final global model parameters are passed to the Reptile stage.

Reptile stage: Unlike the FedAvg stage, the Reptile stage employs the Adam optimizer for local model updates. The aggregation method also differs: instead of sample-size-based weighting, the global server treats each local client as a distinct meta-learning task and applies the Reptile algorithm to compute the combined gradient direction. The global model is then updated with momentum based on this aggregated gradient. After multiple iterations, the final global model parameters are delivered to each local client for the subsequent personalized stage.

Personalized stage: In the Personalize stage, the local clients do not share any data to the global server, and only fine-tune the local models with their own data sets based on the stochastic gradient descent algorithm. And the initial parameters of the local models are the final parameters of the global model of the Reptile stage.

During the whole training process, the raw data of a local client or hospital is never shared with the global server and other local clients, which ensures the security and privacy of the local data. The global server performs aggregation operations on the parameters of the local model so that the local clients can share the training results, effectively avoiding overfitting when the data samples of a single client are too small. The Reptile stage creates ideal conditions for rapid fine-tuning of the local model, and the Personalize stage can effectively solve the problem of data heterogeneity among different hospitals or centers.

**Figure 1:**
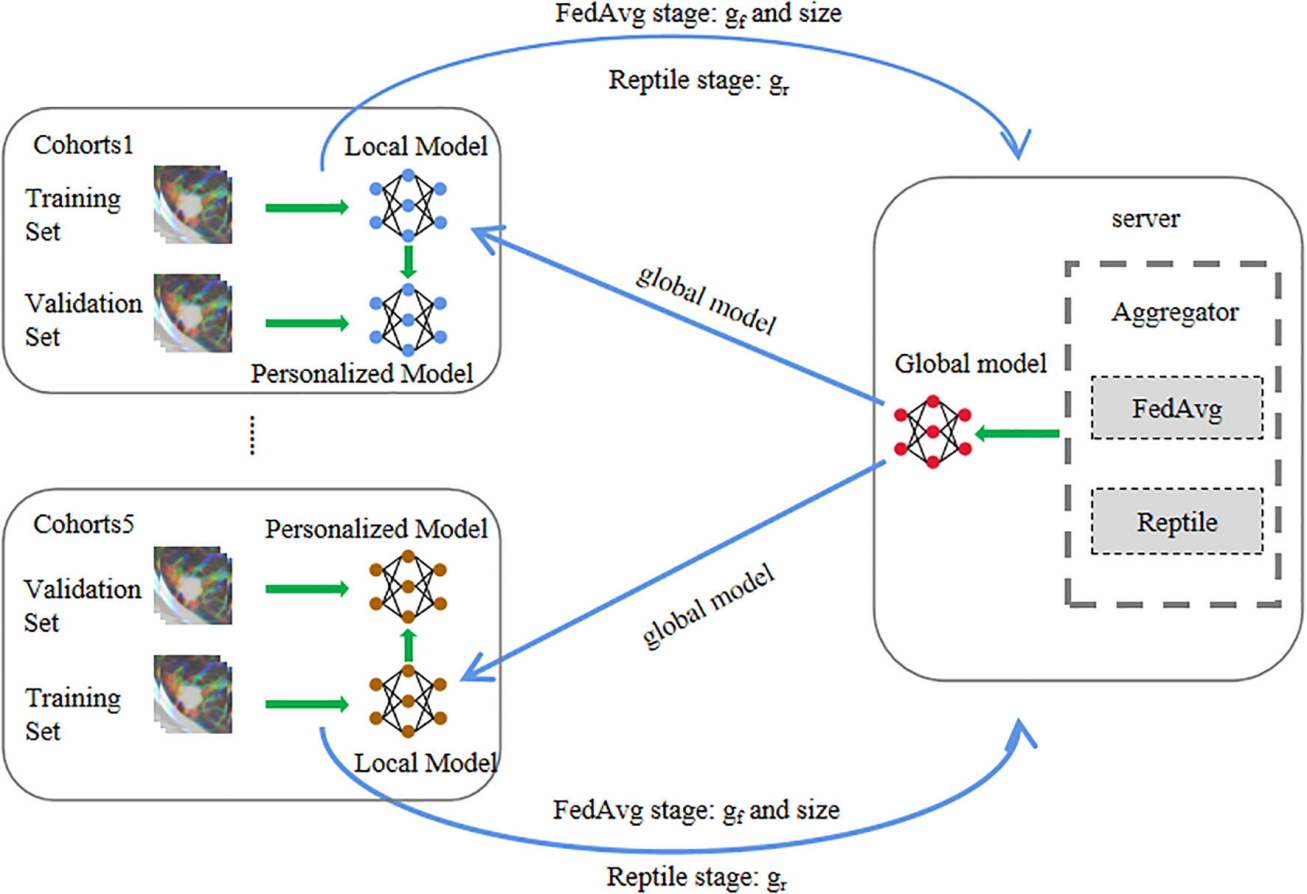
The training process of the meta-learning-based robust federated learning model.

More detailed information regarding the model and training details can be found in **Supplementary S3** and **Figure 1**. The local hospitals utilizes the robust personalized local models, trained by the proposed method, to extract 3904 features from the CT images at each layer of the ROI. Subsequently, the features from all layers are fused (refer to **Supplementary S4** for detailed information).

The classifier can utilize numerous features obtained from the above operations to diagnose TBG or LAC. However, most of these features are not conducive for diagnosing pulmonary nodules and may introduce noise, negatively affecting diagnostic accuracy. Therefore, in the process of feature selection and classifier training, the Mann-Whitney U test is employed to evaluate the diagnostic significance of features, retaining only those with a p-value < 0.05. Finally, a Bayesian extreme learning machine is employed to building the personalized federated learning signature (PFLS) using the selected features (21). To validate its effectiveness, we further performed systematic comparisons with several widely used classifiers, including logistic regression (LR), support vector machine (SVM), and random forest (RF). These models are representative in medical image analysis and AI classification tasks, covering linear, kernel-based, and ensemble learning approaches, respectively. All models were trained and evaluated under identical data splits and preprocessing settings to ensure fair comparison. The pseudocode of the algorithm is provided in **Supplementary S5**.

### Personalized federated learning signature comparison FedAvg model

The FedAvg (22) model is the federated learning model and the global model trained through the federated averaging algorithm. In the training iteration of FedAvg, each client accepts the global model parameters, initiating local training based on this global model. After training by the local clients on local data, the parameters of the local models are uploaded, and the parameters of each local model are averagely weighted by the global server to achieve collaborative training of the ResNet18 global model. More model parameter Settings can be found in **Supplementary S6**. Subsequently, each local hospital uses the same global model to extract 3904 features from the CT images, followed by utilization of Mann-Whitney U test to select features with significant difference from the extracted features. Finally, a Bayesian Extreme Learning Machine is employed for classification.

### Personalized federated learning signature comparison independent local models

Independent local models(ILM) are the ResNet18 and trained exclusively with local data, with no data interaction occurring among the local models from other hospitals. The training process of this model first involves pre-training using ImageNet data, and then training respective models with each local dataset. More model parameter Settings can be found in **Supplementary S5**. Through the ResNet18 model, 3904 deep learning features are extracted from the CT image of each case. Features with significant differences are identified using the Mann-Whitney U test. Eventually, a Bayesian Extreme Learning Machine is applied to perform classification using these selected features.

### Personalized federated learning signature comparison building the clinical model

CT image data are collected from a total of five cohorts, with data from each hospital divided into training and test sets. The patient distribution and clinical features of the CT images are outlined in **Table 1**. Thus, this study selects clinical features (gender, age, nodule size, shape of lesion margin, lobulated shape, and spiculated sign to build the clinical model(CM) based on Bayesian Extreme Learning Machine.

**Table 1:** The patient distribution and clinical features.

Clinical information	Cohort 1						Cohort 2						Cohort 3					
	Training set (n=161)			Testing set (n=109)			Training set (n=51)			Testing set (n=36)			Training set (n=70)			Testing set (n=49)		
	TBG	LAC	P value	TBG	LAC	P value	TBG	LAC	P valule	TBG	LAC	P valule	TBG	LAC	P valule	TBG	LAC	P valule
	(n=34)	(n=127)		(n=23)	(n=86)		(n=14)	(n=37)		(n=10)	(n=26)		(n=21)	(n=49)		(n=15)	(n=34)	
Gender																		
Male	21	57	0.08	12	39	0.56	9	20	0.51	5	16	0.529	14	25	0.227	6	24	0.043
Female	13	70		11	47		5	17		5	10		7	24		9	10	
Age(mean ± SD, years)																		
	59.2±13.8	57.8±10.1	0.533	53.0±11.5	59.8±10.9	0.011	53.8±12.1	59.4±9.3	0.087	61.2±9.2	57.8±12.2	0.433	47.9±15.6	58.7±9.2	0.001	50.7±11.5	60.7±9.7	0.003
Lesion size(mm)																		
	14.5±8.0	20.7±9.7	0.001	12.7±7.4	20.3±9.1	<0.001	18.7±6.00	20.9±13.3	0.555	20.5±7.9	25.8±22.1	0.467	12.1±8.0	14.1±5.1	0.206	12.3±5.6	15.5±4.8	0.046
Location																		
Upper and middle	17	89	0.028	13	56	0.447	10	27	0.912	6	16	0.932	12	26	0.753	9	25	0.344
Lower	17	38		10	30		4	10		4	10		9	23		6	9	
Lesion Margin																		
Irregular	20	117	<0.001	16	71	0.168	12	33	0.731	8	24	0.293	11	42	0.003	9	33	0.001
Regular	14	10		7	15		2	4		2	2		10	7		6	1	
Lobulated shape																		
Absence	22	18	<0.001	12	24	0.028	5	4	0.037	3	0	0.004	11	8	0.002	8	1	<0.001
Presence	12	109		11	62		9	33		7	26		10	41		7	33	
Spiculated sign																		
Absence	28	70	0.004	20	51	0.013	10	13	0.02	9	7	0.001	18	27	0.014	14	13	<0.001
Presence	6	57		3	35		4	24		1	19		3	22		1	21	

Clinical information	Cohort 4						Cohort 5											
	Training Set (n=282)			Testing Set (n=189)			Training Set (n=46)			Testing Set (n=33)								
	TBG	LAC	p valule	TBG	LAC	p valule	TBG	LAC	p valule	TBG	LAC	p valule						
	(n=96)	(n=186)		(n=64)	(n=125)		(n=22)	(n=24)		(n=16)	(n=17)							
Gender																		
Male	61	93	0.03	42	53	0.003	13	11	0.369	10	7	0.221						
Female	35	93		22	72		9	13		6	10							
Age(mean ± SD, years)																		
	51.4±12.9	60.8±9.8	<0.001	52.3±11.3	60.5±10.7	<0.001	53.3±9.2	63.1±8.6	0.001	47.3±11.9	59.8±9.9	0.002						
Lesion size(mm)																		
	12.3±6.6	17.6±8.2	<0.001	13.1±7.5	17.4±8.5	0.001	10.0±6.4	17.3±9.8	0.005	8.6±3.8	25.1±25.4	0.015						
Location																		
Upper and middle	65	125	0.932	44	80	0.515	11	16	0.251	8	14	0.049						
Lower	31	61		20	45		11	8		8	3							
Lesion Margin																		
Irregular	53	160	<0.001	35	109	<0.001	14	22	0.021	6	17	<0.001						
Regular	43	26		29	16		8	2		10	0							
Lobulated shape																		
Absence	60	32	<0.001	37	21	<0.001	11	12	1	12	5	0.009						
Presence	36	154		27	104		11	12		4	12							
Spiculated sign																		
Absence	80	98	<0.001	55	65	<0.001	12	14	0.796	13	11	0.286						
Presence	16	88		9	60		10	10		3	6							

### Personalized federated learning signature comparison merged data centralized model

To validate the necessity and advantages of the proposed Personalized Federated Learning Signature (PFLS) framework, we established a Merged Data Centralized Model (MDCM) as a comparative benchmark. This model integrates training data from all participating centers to train a single deep learning model without any privacy constraints—simulating an ideal scenario where data sharing faces no regulatory or ethical barriers. After training, the centralized MDCM was independently evaluated on the local test sets of each hospital to assess its generalization performance across heterogeneous data distributions. This approach enables a quantitative comparison between the centrally trained model and the personalized federated models, highlighting the impact of data heterogeneity and demonstrating the effectiveness of federated learning in maintaining model performance while preserving data privacy.

### Personalized federated learning signature comparison with personalized federated model

To further evaluate the effectiveness of the proposed PFLS framework, we selected several representative personalized federated learning methods for comparison, including FedProx (23), FedBN (24), and Moon (25). FedProx introduces a proximal term into the local objective function to constrain local updates from deviating excessively from the global model, thereby stabilizing the optimization process under non-IID data distributions. FedBN retains the Batch Normalization (BN) parameters locally while aggregating the remaining parameters globally, which alleviates performance degradation caused by feature distribution shifts across centers. Moon incorporates a contrastive learning objective during local training to encourage consistency between local and global representations, thus improving robustness in heterogeneous data scenarios. After federated training, each method employed its respective personalized model to extract features from the local data. Finally, a Bayesian Extreme Learning Machine is employed for classification.

### Ablation experiments on PFLS

To quantitatively verify the effectiveness of the Reptile step (26) and the personalization step in PFLS, we designed ablation experiments. Specifically, we constructed different algorithm variants by selectively removing these two steps: (1) removing the Reptile step while retaining the personalization step, where each site was validated using its own personalized model; (2) removing the personalization step while retaining the Reptile update, where all centers were validated using the global model after the Reptile update. All variants were trained and evaluated under the same experimental settings. By comparing their performance with the complete PFLS, we were able to assess the contribution of each component.

### Statistical analysis

The performance evaluation of the models involved calculating various metrics, including the receiver operating characteristic curve (ROC), area under the curve (AUC), sensitivity, specificity, accuracy, positive probability value (PPV), and negative probability value (NPV). The net reclassification improvement (NRI) and integrated discrimination improvement (IDI) were used to measure the degree of improvement of PFLS in overall discriminative ability compared with FedAvg, ILM, and CM. P-values less than 0.05 were considered a significant difference.

## Results

### Clinical factors and subjective CT findings analysis

The patient distribution and clinical features of the CT images are outlined in **Table 1**.The table details various clinical parameters such as gender, age, lesion size, location, margin, lobulated shape, and spiculated sign, with a clear distinction between training and testing sets within each cohort. A notable observation is the inconsistent distribution of these clinical features across different cohorts. For instance, the proportion of males and females varies significantly, with some cohorts having a higher male prevalence (e.g., Cohort 1 and Cohort 4) while others show a more balanced or female-dominant distribution (e.g., Cohort 3). Similarly, the distribution of lesion location, margin, lobulated shape, and spiculated sign further underscores the heterogeneity among cohorts. For example, the presence of lobulated and spiculated lesions varies widely, suggesting differences in disease characteristics or diagnostic practices across cohorts.

### The performance of PFLS identifies LAC and TBG

**Figure 2:**
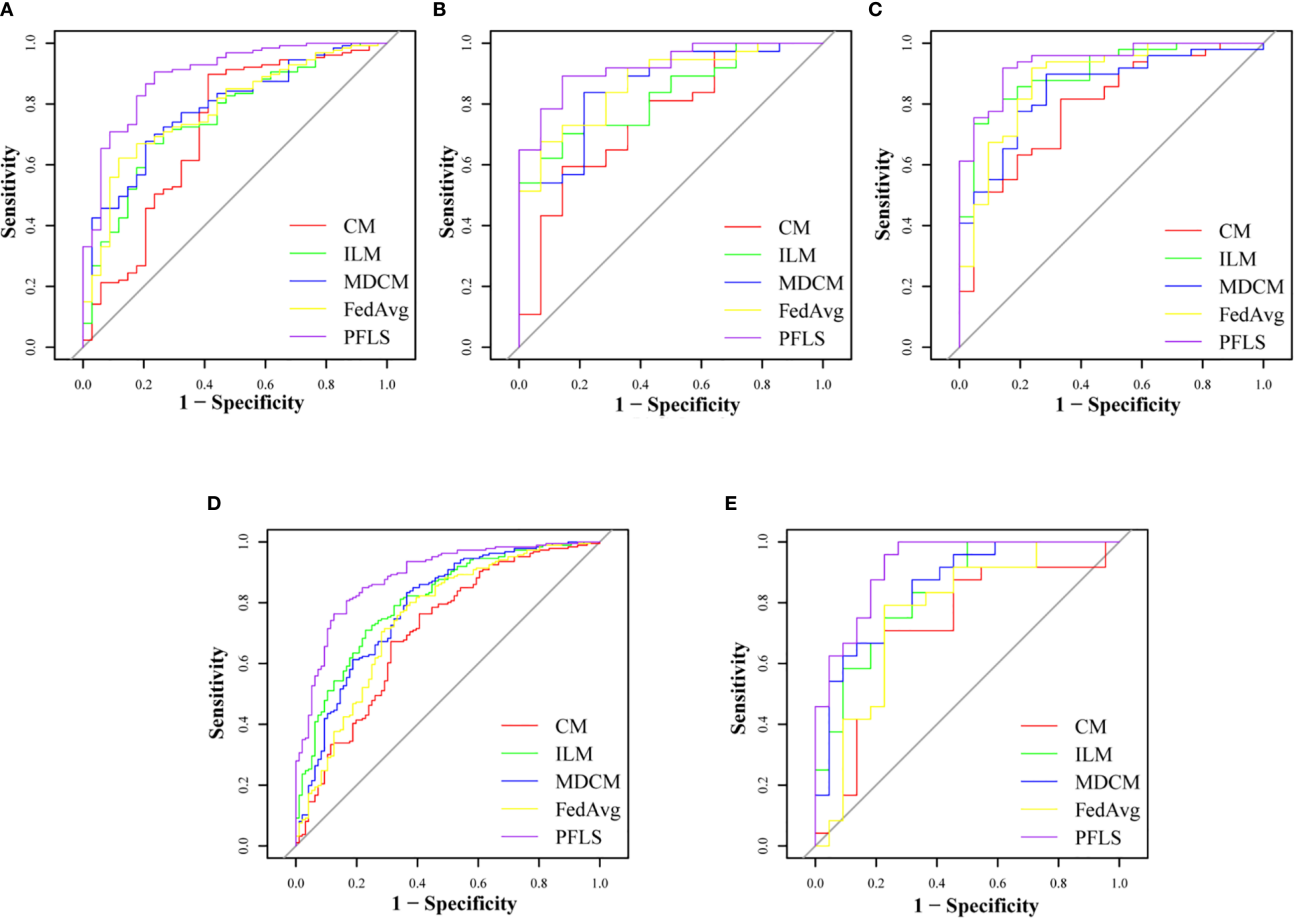
ROC of training set of five cohorts. **(A)** Cohort 1, **(B)** Cohort 2, **(C)** Cohort 3, **(D)** Cohort 4, **(E)** Cohort 5.

**Figure 3:**
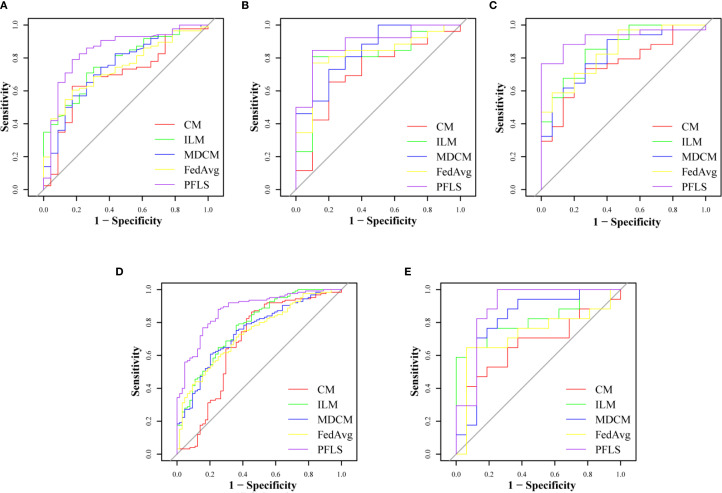
ROC of test set of five cohorts. **(A)** Cohort 1, **(B)** Cohort 2, **(C)** Cohort 3, **(D)** Cohort 4, **(E)** Cohort 5.

**Figure 4:**
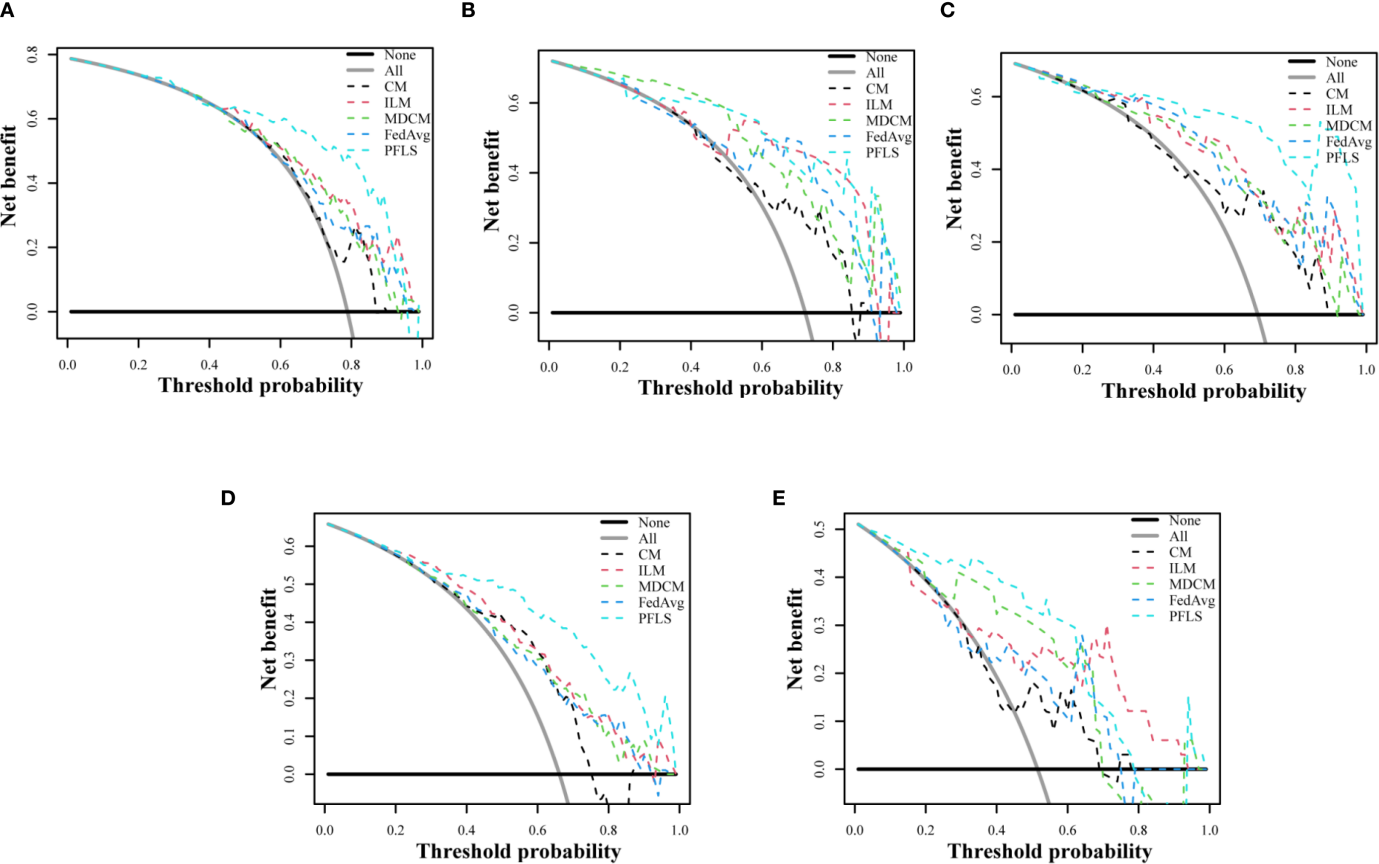
Decision curve analysis of test set of five cohorts. **(A)** Cohort 1, **(B)** Cohort 2, **(C)** Cohort 3, **(D)** Cohort 4, **(E)** Cohort 5.

As shown in **Table 2**, all models were trained and validated using the same feature set to ensure fairness in comparison. The results revealed that SVM and RF experienced severe overfitting during training, as indicated by the large performance gap between the training and test sets. In contrast, logistic regression (LR) did not show obvious overfitting; however, its classification performance was still inferior to that of the Bayesian extreme learning machine (Bayesian ELM). By comparison, Bayesian ELM not only maintained high training efficiency but also demonstrated stronger generalization ability on the test set. The performance of the personalized federated learning signature (PFLS) across the five cohorts is presented in **Figures 2**, **3** and **Table 3**. On the test sets, PFLS achieved AUCs of 0.846 (95% CI, 0.748–0.944), 0.889 (95% CI, 0.771–1.000), 0.922 (95% CI, 0.845–0.999), 0.876 (95% CI, 0.825–0.927), and 0.893 (95% CI, 0.770–1.000), with corresponding accuracies of 0.807, 0.861, 0.878, 0.788, and 0.818. These results indicate that PFLS consistently demonstrated excellent predictive performance across different cohorts, effectively distinguishing lung adenocarcinoma from tuberculosis granulomas. Furthermore, decision curve analysis (**Figure 4**) showed that PFLS provided a higher net benefit than other models in cohorts 2, 3, and 5. **Supplementary Table S1** in the supplementary materials for model details.

**Table 2:** Performance of logistic regression, SVM, random forest and ELM.

Cohort 1	Training set 1				Testing set 1			
	SVM	RF	LR	ELM	SVM	RF	LR	ELM
AUC (95%CI)	0.923 (0.867-0.980)	0.952 (0.920-0.984)	0.822 (0.741-0.903)	0.895 (0.833-0.956)	0.812 (0.700-0.923)	0.852 (0.756-0.949)	0.777 (0.662-0.893)	0.846 (0.748-0.944)
Sensitivity	0.819 (104/127)	0.882 (112/127)	0.724 (92/127)	0.906 (115/127)	0.694 (59/85)	0.800 (68/85)	0.682 (58/85)	0.826 (71/85)
Specificity	0.941 (32/34)	0.941 (32/34)	0.765 (26/34)	0.765 (26/34)	0.792 (19/24)	0.708 (17/24)	0.750 (18/24)	0.739 (17/24)
Accuracy	0.845 (136/161)	0.894 (144/161)	0.733 (118/161)	0.876 (141/161)	0.716 (78/109)	0.780 (85/109)	0.697 (76/109)	0.807 (88/109)
PPV	0.981 (104/106)	0.982 (112/114)	0.920 (92/100)	0.935 (115/123)	0.922 (59/64)	0.907 (68/75)	0.906 (58/64)	0.922 (71/77)
NPV	0.582 (32/55)	0.681 (32/47)	0.426 (26/61)	0.684 (26/38)	0.422 (19/45)	0.500 (17/34)	0.400 (18/45)	0.531 (17/32)

Cohort 2	Training set 2				Testing set 2			
	SVM	RF	LR	ELM	SVM	RF	LR	ELM
AUC (95%CI)	0.842 (0.702-0.981)	0.892 (0.803-0.981)	0.838 (0.729-0.947)	0.925 (0.853-0.997)	0.746 (0.570-0.922)	0.900 (0.796-1.000)	0.831 (0.689-0.973)	0.889 (0.771-1.000)
Sensitivity	0.757 (28/37)	0.757 (28/37)	0.676 (25/37)	0.892 (33/37)	0.808 (21/26)	0.923 (24/26)	0.808 (21/26)	0.923 (24/26)
Specificity	0.929 (13/14)	1.000 (14/14)	0.929 (13/14)	0.786 (11/14)	0.400 (4/10)	0.600 (6/10)	0.600 (6/10)	0.700 (7/10)
Accuracy	0.804 (41/51)	0.824 (42/51)	0.745 (38/51)	0.863 (44/51)	0.694 (25/36)	0.833 (30/36)	0.750 (27/36)	0.861 (31/36)
PPV	0.966 (28/29)	1.000 (28/28)	0.962 (25/26)	0.917 (33/36)	0.778 (21/27)	0.857 (24/28)	0.840 (21/25)	0.889 (24/27)
NPV	0.591 (13/22)	0.609 (14/23)	0.520 (13/25)	0.733 (11/15)	0.444 (4/9)	0.750 (6/8)	0.545 (6/11)	0.778 (7/9)

Cohort 3	Training set 3				Testing set 3			
	SVM	RF	LR	ELM	SVM	RF	LR	ELM
AUC (95%CI)	0.964 (0.924-1.000)	0.984 (0.948-1.000)	0.832 (0.707-0.957)	0.939 (0.884-0.994)	0.888 (0.784-0.992)	0.953 (0.897-1.000)	0.790 (0.620-0.960)	0.922 (0.845-0.999)
Sensitivity	0.939 (46/49)	0.959 (47/49)	0.857 (42/49)	0.918 (45/49)	0.794 (27/34)	0.971 (33/34)	0.824 (28/34)	0.882 (30/34)
Specificity	0.952 (20/21)	1.000 (21/21)	0.762 (16/21)	0.857 (18/21)	0.800 (12/15)	0.733 (11/15)	0.800 (12/15)	0.867 (13/15)
Accuracy	0.943 (66/70)	0.971 (68/70)	0.829 (58/70)	0.900 (63/70)	0.796 (39/49)	0.898 (44/49)	0.816 (40/49)	0.878 (43/49)
PPV	0.979 (46/47)	1.000 (47/47)	0.894 (42/47)	0.938 (45/48)	0.900 (27/30)	0.892 (33/37)	0.903 (28/31)	0.938 (30/32)
NPV	0.870 (20/23)	0.913 (21/23)	0.696 (16/23)	0.818 (18/22)	0.632 (12/19)	0.917 (11/12)	0.667 (12/18)	0.765 (13/17)

Cohort 4	Training set 4				Testing set 4			
	SVM	RF	LR	ELM	SVM	RF	LR	ELM
AUC (95%CI)	0.931 (0.903-0.960)	0.972 (0.956-0.987)	0.837 (0.786-0.888)	0.886 (0.845-0.926)	0.869 (0.814-0.923)	0.875 (0.819-0.931)	0.834 (0.769-0.9000)	0.876 (0.825-0.927)
Sensitivity	0.812 (151/186)	0.930 (173/186)	0.726 (135/186)	0.807 (150/186)	0.736 (92/125)	0.824 (103/125)	0.736 (92/125)	0.768 (96/125)
Specificity	0.906 (87/96)	0.896 (86/96)	0.833 (80/96)	0.833 (80/96)	0.828 (53/64)	0.766 (49/64)	0.797 (51/64)	0.828 (53/64)
Accuracy	0.844 (238/282)	0.918 (259/282)	0.762 (215/282)	0.816 (230/282)	0.767 (145/189)	0.804 (152/189)	0.757 (143/189)	0.788 (149/189)
PPV	0.944 (151/160)	0.945 (173/183)	0.894 (135/151)	0.904 (150/166)	0.893 (92/103)	0.873 (103/118)	0.876 (92/105)	0.897 (96/107)
NPV	0.713 (87/122)	0.869 (86/99)	0.611 (80/131)	0.690 (80/116)	0.616 (53/86)	0.690 (49/71)	0.607 (51/84)	0.646 (53/82)

Cohort 5	Training set 5				Testing set 5			
	SVM	RF	LR	ELM	SVM	RF	LR	ELM
AUC (95%CI)	0.886 (0.793-0.980)	0.949 (0.894-1.000)	0.837 (0.724-0.951)	0.924 (0.850-0.998)	0.893 (0.780-1.000)	0.805 (0.656-0.955)	0.831 (0.679-0.983)	0.893 (0.770-1.000)
Sensitivity	0.833 (20/24)	0.875 (21/24)	0.708 (17/24)	0.958 (23/24)	0.882 (15/17)	0.941 (16/17)	0.882 (15/17)	0.824 (14/17)
Specificity	0.864 (19/22)	0.909 (20/22)	0.864 (19/22)	0.727 (16/22)	0.750 (12/16)	0.500 (8/16)	0.750 (12/16)	0.813 (13/16)
Accuracy	0.848 (39/46)	0.891 (41/46)	0.783 (36/46)	0.848 (39/46)	0.818 (27/33)	0.727 (24/33)	0.818 (27/33)	0.818 (27/33)
PPV	0.870 (20/23)	0.913 (21/23)	0.850 (17/20)	0.793 (23/29)	0.789 (15/19)	0.667 (16/24)	0.789 (15/19)	0.824 (14/17)
NPV	0.826 (19/23)	0.870 (20/23)	0.731 (19/26)	0.941 (16/17)	0.857 (12/14)	0.889 (8/9)	0.857 (12/14)	0.813 (13/16)

**Table 3:** Performance of the of models on the each cohort.

Cohort 1	Training set 1					Testing set 1				
	CM	ILM	MDCM	Fedavg	PFLS	CM	ILM	MDCM	Fedavg	PFLS
AUC (95%CI)	0.712 (0.601-0.824)	0.759 (0.669-0.849)	0.784 (0.702-0.866)	0.782 (0.698-0.866)	0.895 (0.833-0.956)	0.692 (0.569-0.815)	0.773 (0.672-0.873)	0.752 (0.630-0.857)	0.740 (0.637-0.843)	0.846 (0.748-0.944)
Sensitivity	0.898 (114/127)	0.669 (85/127)	0.677 (86/127)	0.622 (79/127)	0.906 (115/127)	0.744 (64/86)	0.709 (61/86)	0.588 (50/86)	0.477 (41/86)	0.826 (71/86)
Specificity	0.588 (20/34)	0.794 (27/34)	0.794 (27/34)	0.882 (30/34)	0.765 (26/34)	0.391 (9/23)	0.739 (17/23)	0.750 (18/23)	0.870 (20/23)	0.739 (17/23)
Accuracy	0.832 (134/161)	0.696 (112/161)	0.702 (113/161)	0.677 (109/161)	0.876 (141/161)	0.670 (73/109)	0.716 (78/109)	0.624 (68/109)	0.560 (61/109)	0.807 (88/109)
PPV	0.891 (114/128)	0.924 (85/92)	0.925 (86/93)	0.952 (79/83)	0.935 (115/123)	0.821 (64/78)	0.910 (61/67)	0.893 (50/56)	0.932 (41/44)	0.922 (71/77)
NPV	0.606 (20/33)	0.391 (27/69)	0.397 (27/68)	0.385 (30/78)	0.684 (26/38)	0.290 (9/31)	0.405 (17/42)	0.340 (18/53)	0.308 (20/65)	0.531 (17/32)

Cohort 2	Training set 2					Testing set 2				
	CM	ILM	MDCM	FedAvg	PFLS	CM	ILM	MDCM	FedAvg	PFLS
AUC (95%CI)	0.749 (0.594-0.905)	0.828 (0.715-0.941)	0.855 (0.742-0.968)	0.869 (0.767-0.971)	0.925 (0.853-0.997)	0.719 (0.523-0.915)	0.808 (0.640-0.975)	0.842 (0.698-0.987)	0.823 (0.672-0.974)	0.889 (0.771-1.000)
Sensitivity	0.595 (22/37)	0.703 (26/37)	0.838 (31/37)	0.676 (25/37)	0.892 (33/37)	0.654 (17/26)	0.808 (21/26)	0.885 (23/26)	0.615 (16/26)	0.923 (24/26)
Specificity	0.857 (12/14)	0.857 (12/14)	0.786 (11/14)	0.857 (12/14)	0.786 (11/14)	0.700 (7/10)	0.900 (9/10)	0.600 (6/10)	0.900 (9/10)	0.700 (7/10)
Accuracy	0.667 (34/51)	0.745 (38/51)	0.824 (42/51)	0.726 (37/51)	0.863 (44/51)	0.667 (24/36)	0.833 (30/36)	0.806 (29/36)	0.694 (25/36)	0.861 (31/36)
PPV	0.917 (22/24)	0.929 (26/28)	0.912 (31/34)	0.926 (25/27)	0.917 (33/36)	0.850 (17/20)	0.955 (21/22)	0.852 (23/27)	0.941 (16/17)	0.889 (24/27)
NPV	0.444 (12/27)	0.522 (12/23)	0.647 (11/17)	0.500 (12/24)	0.733 (11/15)	0.438 (7/16)	0.643 (9/14)	0.667 (6/9)	0.474 (9/19)	0.778 (7/9)

Cohort 3	Training set 3					Testing set 3				
	CM	ILM	MDCM	FedAvg	PFLS	CM	ILM	MDCM	FedAvg	PFLS
AUC (95%CI)	0.794 (0.680-0.908)	0.903 (0.829-0.977)	0.847 (0.752-0.943)	0.880 (0.786-0.973)	0.939 (0.884-0.994)	0.759 (0.618-0.900)	0.857 (0.745-0.969)	0.825 (0.701-0.950)	0.839 (0.724-0.955)	0.922 (0.845-0.999)
Sensitivity	0.816 (40/49)	0.735 (36/49)	0.898 (44/49)	0.918 (45/49)	0.918 (45/49)	0.882 (30/34)	0.735 (25/34)	0.824 (28/34)	0.971 (33/34)	0.882 (30/34)
Specificity	0.667 (14/21)	0.905 (19/21)	0.714 (15/21)	0.714 (15/21)	0.857 (18/21)	0.333 (5/15)	0.733 (11/15)	0.600 (9/15)	0.533 (8/15)	0.867 (13/15)
Accuracy	0.771 (54/70)	0.786 (55/70)	0.843 (59/70)	0.857 (60/70)	0.900 (63/70)	0.714 (35/49)	0.735 (36/49)	0.755 (37/49)	0.837 (41/49)	0.878 (43/49)
PPV	0.851 (40/47)	0.947 (36/38)	0.880 (44/50)	0.882 (45/51)	0.938 (45/48)	0.750 (30/40)	0.862 (25/29)	0.824 (28/34)	0.825 (33/40)	0.938 (30/32)
NPV	0.609 (14/23)	0.594 (19/32)	0.750 (15/20)	0.790 (15/19)	0.818 (18/22)	0.556 (5/9)	0.550 (11/20)	0.600 (9/15)	0.889 (8/9)	0.765 (13/17)

Cohort 4	Training set 4					Testing set 4				
	CM	ILM	MDCM	FedAvg	PFLS	CM	ILM	MDCM	FedAvg	PFLS
AUC (95%CI)	0.706 (0.639-0.772)	0.805 (0.751-0.858)	0.785 (0.727-0.844)	0.748 (0.685-0.812)	0.886 (0.845-0.926)	0.679 (0.587-0.770)	0.778 (0.707-0.848)	0.746 (0.673-0.818)	0.737 (0.664-0.810)	0.876 (0.825-0.927)
Sensitivity	0.672 (125/186)	0.710 (132/186)	0.833 (155/186)	0.801 (149/186)	0.807 (150/186)	0.664 (83/125)	0.720 (90/125)	0.768 (96/125)	0.752 (94/125)	0.768 (96/125)
Specificity	0.688 (66/96)	0.771 (74/96)	0.635 (61/96)	0.615 (59/96)	0.833 (80/96)	0.641 (41/64)	0.656 (42/64)	0.594 (38/64)	0.594 (38/64)	0.828 (53/64)
Accuracy	0.677 (191/282)	0.731 (206/282)	0.766 (216/282)	0.738 (208/282)	0.816 (230/282)	0.656 (124/189)	0.698 (132/189)	0.709 (134/189)	0.698 (132/189)	0.788 (149/189)
PPV	0.806 (125/155)	0.857 (132/154)	0.816 (155/190)	0.801 (149/186)	0.904 (150/166)	0.783 (83/106)	0.804 (90/112)	0.787 (96/122)	0.783 (94/120)	0.897 (96/107)
NPV	0.520 (66/127)	0.578 (74/128)	0.663 (61/92)	0.615 (59/96)	0.690 (80/116)	0.494 (41/83)	0.546 (42/77)	0.567 (38/67)	0.551 (38/69)	0.646 (53/82)

Cohort 5	Training set 5					Testing set 5				
	CM	ILM	MDCM	FedAvg	PFLS	CM	ILM	MDCM	FedAvg	PFLS
AUC (95%CI)	0.714 (0.555-0.873)	0.835 (0.720-0.951)	0.854 (0.744-0.964)	0.769 (0.623-0.915)	0.924 (0.850-0.998)	0.665 (0.471-0.860)	0.824 (0.676-0.972)	0.820 (0.661-0.979)	0.728 (0.537-0.919)	0.893 (0.770-1.000)
Sensitivity	0.708 (17/24)	0.750 (18/24)	0.792 (19/24)	0.792 (19/24)	0.958 (23/24)	0.647 (11/17)	0.882 (15/17)	0.412 (7/17)	0.647 (11/17)	0.824 (14/17)
Specificity	0.773 (17/22)	0.727 (16/22)	0.773 (17/22)	0.773 (17/22)	0.727 (16/22)	0.625 (10/16)	0.375 (6/16)	0.875 (14/16)	0.688 (11/16)	0.813 (13/16)
Accuracy	0.739 (34/46)	0.739 (34/46)	0.783 (36/46)	0.783 (36/46)	0.848 (39/46)	0.636 (21/33)	0.636 (21/33)	0.636 (21/33)	0.667 (22/33)	0.818 (27/33)
PPV	0.773 (17/22)	0.750 (18/24)	0.792 (19/24)	0.792 (19/24)	0.793 (23/29)	0.647 (11/17)	0.600 (15/25)	0.778 (7/9)	0.688 (11/16)	0.824 (14/17)
NPV	0.708 (17/24)	0.727 (16/22)	0.773 (17/22)	0.773 (17/22)	0.941 (16/17)	0.625 (10/16)	0.750 (6/8)	0.583 (14/24)	0.647 (11/17)	0.813 (13/16)

### Comparison of the PFLS with the FedAvg model

As shown in **Table 3**, the AUC of the FedAvg model on the test sets from the five cohorts are 0.740 (95% CI, 0.637-0.843), 0.823 (95% CI, 0.672-0.974), 0.839 (95% CI, 0.724-0.955), 0.737 (95% CI, 0.664-0.810), and 0.728 (95% CI, 0.537-0.919). The FedAvg model achieved accuracy of 0.560, 0.694, 0.837, 0.698, and 0.667 on the test sets of each cohort. Compared with FedAvg, the AUC of PFLS at each cohort increased by 6.6%-16.5%, and the accuracy rate increased by 4.1%-25.1%. **Supplementary Table S2** and **Supplementary Table S3** respectively present the results of NRI and IDI. The results of NRI and IDI show that the performance of PFLS on each central test set is improved compared with the FedAvg model. The range values of NRI at each cohort were 0.287 to 1.263. The range values of IDI at each cohort were 0.016 to 0.069. **Supplementary Table S4** in the supplementary materials for FedAvg model details.

### Comparison of the PFLS with ILM

The AUC of ILM on the test sets from each cohort are 0.773(95% CI, 0.672-0.873), 0.808 (95% CI, 0.640-0.975), 0.857 (95% CI, 0.745-0.969), 0.778 (95% CI, 0.707-0.848), and 0.824 (95% CI, 0.676-0.972). The ILM achieved accuracy of 0.716, 0.833, 0.735, 0.698, and 0.636 on the test sets of each cohort. Compared to ILM, the PFLS achieved an AUC improvement of 6.5%- 9.4% across the cohorts, and the PFLS showed an improvement of 2.8%-19.1% in the prediction accuracy for LAC and TBG at each cohort. More details are provided in **Table 3** and **Figure 2**, **3**. As shown in **Supplementary Table S2** and **Supplementary Table S3**, the performance of PFLS on each central test set is improved compared with the ILM. The range values of NRI at each cohort were 0.324 to 969. The range values of IDI at each cohort were 0.022 to 0.061. **Supplementary Table S5** in the supplementary materials for ILM details.

### Comparison of the PFLS with the CM

As shown in **Table 3**, the AUC of the CMmodel on the test sets from the five cohorts are 0.692(95% CI, 0.569-0.815), 0.719(95% CI, 0.523-0.915), 0.759(95% CI, 0.618-0.900), 0.679(95% CI, 0.587-0.770), and 0.665(95% CI, 0.471-0.860). In comparison, the experimental results indicate that the PFLS outperforms the CM in all metrics. As shown in **Supplementary Table S2**, **Supplementary Table S3**, in the test sets, PFLS exhibits higher NRI and IDI indices with significant p-values (p < 0.05) compared to CM across cohorts, indicating its superiority.

### Comparison of the PFLS with MDCM

As shown in **Table 3**, the AUC of the MDCM on the test sets from the five cohorts are 0.752, 0.842, 0.825, 0.746, and 0.820. The experimental results indicate that the MDCM exhibits relatively poor consistency in performance across different centers. This performance variability may be attributed to the inherent heterogeneity in the data from various centers. When training on merged data, such heterogeneity can lead to suboptimal model generalization, as the model may be biased toward certain center-specific characteristics rather than capturing universally representative features. Consequently, the model’s ability to perform consistently well across diverse and unseen datasets is compromised.

### Comparison of the PFLS with personalized federated model

As shown in **Table 4**, the three personalized federated models, FedProx, FedBN, and Moon, achieved AUC values ranging from 0.746 to 0.834 and accuracies between 0.619 and 0.749 across the five cohorts. However, all three methods exhibited unstable performance when confronted with heterogeneous multi-center data. In contrast, our proposed PFLS consistently outperformed them, with AUC improvements of approximately 4%-15% and accuracy gains of 6%-22% across different cohorts. The performance of PFLS and other personalized federated learning algorithms on the training and test sets across the five cohorts is shown in **Supplementary Figure S2**, **Supplementary Figure S3**. Additionally, the Decision Curve Analysis are shown in **Supplementary Figure S4**.

**Table 4:** Performance comparison of models trained under different FL algorithms.

Cohort 1	Training set 1				Testing set 1			
	Fedprox	FedBN	Moon	PFLS	Fedprox	FedBN	Moon	PFLS
AUC (95%CI)	0.790 (0.716-0.863)	0.770 (0.677-0.863)	0.761 (0.671-0.851)	0.895 (0.833-0.956)	0.764 (0.646-0.883)	0.741 (0.628-0.853)	0.752 (0.639-0.865)	0.846 (0.748-0.944)
Sensitivity	0.567 (72/127)	0.835 (106/127)	0.512 (65/127)	0.906 (115/127)	0.512 (44/86)	0.651 (56/86)	0.523 (45/86)	0.826 (71/86)
Specificity	0.912 (31/34)	0.618 (21/34)	0.912 (31/34)	0.765 (26/34)	0.870 (20/23)	0.739 (17/23)	0.783 (18/23)	0.739 (17/23)
Accuracy	0.640 (103/161)	0.789 (127/161)	0.596 (96/161)	0.876 (141/161)	0.587 (64/109)	0.670 (73/109)	0.578 (63/109)	0.807 (88/109)
PPV	0.960 (72/75)	0.891 (106/119)	0.956 (65/68)	0.935 (115/123)	0.936 (44/47)	0.903 (56/62)	0.900 (45/50)	0.922 (71/77)
NPV	0.360 (31/86)	0.500 (21/42)	0.333 (31/93)	0.684 (26/38)	0.323 (20/62)	0.362 (17/47)	0.305 (18/59)	0.531 (17/32)

Cohort 2	Training set 2				Testing set 2			
	FedProx	FedBN	Moon	PFLS	FedProx	FedBN	Moon	PFLS
AUC (95%CI)	0.844 (0.729-0.959)	0.849 (0.735-0.965)	0.842 (0.717-0.970)	0.925 (0.853-0.997)	0.800 (0.610-0.990)	0.808 (0.620-0.991)	0.813 (0.648-0.967)	0.889 (0.771-1.000)
Sensitivity	0.784 (29/37)	0.757 (28/37)	0.946 (35/37)	0.892 (33/37)	0.654 (17/26)	0.962 (25/26)	0.885 (23/26)	0.923 (24/26)
Specificity	0.857 (12/14)	0.857 (12/14)	0.643 (9/14)	0.786 (11/14)	0.800 (8/10)	0.500 (5/10)	0.600 (6/10)	0.700 (7/10)
Accuracy	0.804 (41/51)	0.784 (40/51)	0.863 (44/51)	0.863 (44/51)	0.694 (25/36)	0.833 (30/36)	0.806 (29/36)	0.861 (31/36)
PPV	0.935 (29/31)	0.933 (28/30)	0.875 (35/40)	0.917 (33/36)	0.895 (17/19)	0.833 (25/30)	0.852 (23/27)	0.889 (24/27)
NPV	0.600 (12/20)	0.571 (12/21)	0.818 (9/11)	0.733 (11/15)	0.471 (8/17)	0.833 (5/6)	0.667 (6/9)	0.778 (7/9)

Cohort 3	Training set 3				Testing set 3			
	FedProx	FedBN	Moon	PFLS	FedProx	FedBN	Moon	PFLS
AUC (95%CI)	0.875 (0.772-0.977)	0.847 (0.753-0.945)	0.869 (0.758-0.980)	0.939 (0.884-0.994)	0.831 (0.701-0.961)	0.812 (0.664-0.960)	0.829 (0.665-0.994)	0.922 (0.845-0.999)
Sensitivity	0.776 (38/49)	0.796 (39/49)	0.776 (38/49)	0.918 (45/49)	0.824 (28/34)	0.853 (29/34)	0.794 (27/34)	0.882 (30/34)
Specificity	0.905 (19/21)	0.857 (18/21)	0.905 (19/21)	0.857 (18/21)	0.733 (11/15)	0.667 (10/15)	0.800 (12/15)	0.867 (13/15)
Accuracy	0.814 (57/70)	0.814 (57/70)	0.814 (57/70)	0.900 (63/70)	0.796 (39/49)	0.796 (39/49)	0.796 (39/49)	0.878 (43/49)
PPV	0.950 (38/40)	0.929 (39/42)	0.950 (38/40)	0.938 (45/48)	0.875 (28/32)	0.853 (29/34)	0.900 (27/30)	0.938 (30/32)
NPV	0.633 (19/30)	0.643 (18/28)	0.633 (19/30)	0.818 (18/22)	0.647 (11/17)	0.667 (10/15)	0.632 (12/19)	0.765 (13/17)

Cohort 4	Training set 4				Testing set 4			
	FedProx	FedBN	Moon	PFLS	FedProx	FedBN	Moon	PFLS
AUC (95%CI)	0.779 (0.723-0.834)	0.754 (0.696-0.813)	0.802 (0.746-0.857)	0.886 (0.845-0.926)	0.748 (0.674-0.822)	0.730 (0.651-0.809)	0.755 (0.683-0.827)	0.876 (0.825-0.927)
Sensitivity	0.672 (125/186)	0.672 (125/186)	0.688 (128/186)	0.807 (150/186)	0.760 (95/125)	0.680 (85/125)	0.648 (81/125)	0.768 (96/125)
Specificity	0.792 (76/96)	0.729 (70/96)	0.813 (78/96)	0.833 (80/96)	0.641 (41/64)	0.672 (43/64)	0.734 (47/64)	0.828 (53/64)
Accuracy	0.713 (201/282)	0.691 (195/282)	0.730 (206/282)	0.816 (230/282)	0.720 (136/189)	0.677 (128/189)	0.677 (128/189)	0.788 (149/189)
PPV	0.862 (125/145)	0.828 (125/151)	0.877 (128/146)	0.904 (150/166)	0.805 (95/118)	0.802 (85/106)	0.827 (81/98)	0.897 (96/107)
NPV	0.555 (76/137)	0.534 (70/131)	0.574 (78/136)	0.690 (80/116)	0.577 (41/71)	0.518 (43/83)	0.516 (47/91)	0.646 (53/82)

Cohort 5	Training set 5				Testing set 5			
	FedProx	FedBN	Moon	PFLS	FedProx	FedBN	Moon	PFLS
AUC (95%CI)	0.845 (0.731-0.958)	0.758 (0.614-0.901)	0.820 (0.698-0.941)	0.924 (0.850-0.998)	0.798 (0.644-0.951)	0.746 (0.571-0.921)	0.809 (0.652-0.962)	0.893 (0.770-1.000)
Sensitivity	0.667 (16/24)	0.667 (16/24)	0.583 (14/24)	0.958 (23/24)	0.471 (8/17)	0.588 (10/17)	0.412 (7/17)	0.824 (14/17)
Specificity	0.909 (20/22)	0.818 (18/22)	0.955 (21/22)	0.727 (16/22)	0.875 (14/16)	0.688 (11/16)	0.875 (14/16)	0.813 (13/16)
Accuracy	0.783 (36/46)	0.739 (34/46)	0.761 (35/46)	0.848 (39/46)	0.667 (22/33)	0.636 (21/33)	0.636 (21/33)	0.818 (27/33)
PPV	0.889 (16/18)	0.800 (16/20)	0.933 (14/15)	0.793 (23/29)	0.800 (8/10)	0.667 (10/15)	0.778 (7/9)	0.824 (14/17)
NPV	0.714 (20/28)	0.692 (18/26)	0.677 (21/31)	0.941 (16/17)	0.609 (14/23)	0.611 (11/18)	0.583 (14/24)	0.813 (13/16)

### Ablation experiments

As shown in **Table 5**, we present the AUC values on the training and test sets for different ablation variants. When the Reptile step was removed, the performance across centers showed little variation but remained at a relatively low level, indicating that the absence of meta-update limited the global model’s ability to provide a good initialization. When the personalization step was removed, some centers achieved relatively good results, while others showed significantly lower AUC values, leading to large performance discrepancies across sites. This suggests that without personalization, relying solely on the global model cannot effectively adapt to heterogeneous data distributions. In contrast, the complete PFLS achieved both the best overall performance and balanced results across centers, further demonstrating the complementary roles of the Reptile step and the personalization step in enhancing model generalization and adaptability.

**Table 5:** AUC results of ablation experiments.

Fedavg stage	Reptile stage	Personalization stage	AUC (95%CI)									
			Cohort 1		Cohort 2		Cohort 3		Cohort 4		Cohort 5	
			Train set	Test set	Train set	Test set	Train set	Test set	Train set	Test set	Train set	Test set
✓		✓	0.781 (0.698-0.865)	0.753 (0.650-0.857)	0.851 (0.743-0.960)	0.812 (0.671-0.953)	0.828 (0.718-0.938)	0.812 (0.691-0.933)	0.811 (0.756-0.867)	0.768 (0.696-0.842)	0.845 (0.735-0.955)	0.816 (0.653-0.980)
✓	✓		0.770 (0.685-0.856)	0.740 (0.641-0.839)	0.826 (0.693-0.960)	0.804 (0.620-0.988)	0.832 (0.717-0.947)	0.808 (0.657-0.959)	0.738 (0.677-0.800)	0.698 (0.622-0.775)	0.782 (0.647-0.918)	0.735 (0.562-0.909)
✓	✓	✓	0.895 (0.833-0.956)	0.846 (0.748-0.944)	0.925 (0.853-0.997)	0.889 (0.771-1.000)	0.939 (0.884-0.994)	0.922 (0.845-0.999)	0.886 (0.845-0.926)	0.876 (0.825-0.927)	0.924 (0.850-0.998)	0.893 (0.770-1.000)

## Discussion

The analysis of lung CT images to accurately differentiate between patients with TBG and LAC in a non-invasive manner holds considerable clinical value. In this study, a PFLS is used to collaboratively use CT image data of TBG and LAC from five cohorts while maintaining patient privacy, enabling the training of robust models for each medical institution. The proposed method shows superior predictive performance than the compared methods on the data from each medical institution for distinguishing between TBG and LAC.

Research shows that gender, age, morphology, and spiculation are significantly different between patients with TBG and LAC (27). As malignant tumors predominantly grow in lung parenchyma, nodules of LAC patients are more likely to exhibit irregular margins, lobulation, and spiculation (28). Although lobulation is a distinct feature of malignant lung nodules, several studies have shown that 25% of benign nodules also exhibit lobulation. Spiculation presents a more significant correlation with LAC (29). Pathologically, spiculation is attributed to fibrous tissue proliferation induced by interstitial thickening and peripheral vascular occlusion. However, identification based on morphologic features of nodules is highly subjective, and the criteria for determination vary among radiologists, limiting the utility of shape features in distinguishing between benign and malignant nodules (30). Therefore, the performance of the CM we constructed based on the above features was not satisfactory.

The convolutional neural networks (CNNs) are capable of automatically extracting features from images and generating features at various levels of abstraction. Among CNNs, Low-level layers produce details like edges, textures, and corners, while high-level layers produce globally abstracted features. Deep CNNs are extensively applied in medical imaging and achieving commendable results (31–33). Nevertheless, deep CNNs are susceptible to overfitting, particularly when training with a limited number of samples. Therefore, when training the ILM, we used the transfer learning strategy to pre-train the model with Imagnet data, and then fine-tune the model with the CT data of this study to alleviate the problem of overfitting. However, perhaps due to the smaller number of training samples, the performance of ILM on the test sets of various medical institutions is generally worse than that of PFLS.

Federated learning is a distributed machine learning approach that enables collaborative training of machine learning models using data from multiple hospitals, while eliminating data leakage. It efficiently addresses the data island issue and mitigates model overfitting due to insufficient training samples from a single medical institution. However, due to differences of CT equipment between hospitals, imaging results from different hospitals are heterogeneous, meaning that using the same machine learning model to infer CT images from different hospitals does not ensure satisfactory performance for all hospitals. Except for the slightly better performance of fedag on the test set of institution 2 compared to ILM, the performance of other institutions was worse than that of ILM. This is primarily due to the apparent heterogeneity of the data across hospitals, resulting in apparent discrepancies between the global model and the actual optimal model of a local hospital, and then leading to poorer predictive performance of the global model in certain hospitals. In addition, we also compared our framework with a centralized model (MDCM) trained by directly merging data from all centers. Although this setting represents an ideal scenario without privacy constraints, the MDCM exhibited unstable performance and large variations across sites. This inconsistency can be attributed to the substantial heterogeneity in imaging protocols, scanner vendors, and patient populations among different hospitals. When data are simply pooled, the model may become biased toward dominant site-specific features, thereby limiting its generalizability. In contrast, PFLS achieved more stable and consistent performance across centers, further underscoring the necessity of federated and personalized federated strategies for real-world multi-center applications.

The proposed PFLS employs the Reptile algorithm by treating the local models of federated learning as the different task of meta learning to collaboratively train the global model, and then fine-tunes global models for personalizing local model of each medical institution. The meta-learning-based federated learning frameworks of the proposed method effectively alleviates overfitting. And the robust personalized local models, which are fine-tuned global models by local hospitals, enhance the generalization ability of the local models while effectively mitigating the performance decrease caused by data heterogeneity among different hospitals. In terms of classification performance, the proposed method shows remarkable advantages on all datasets, except for slightly lower AUC values compare to ILM, FedAvg, and CM. NRI and IDI show that, except for medical institutions 2 and 5 with relatively less test data, the PFLS performance of other institutions has significantly improved compared with ILM, FedAvg, and CM.

Compared with traditional machine learning models, Bayesian ELM has significant advantages in alleviating overfitting and balancing model complexity with generalization ability, thereby providing more robust and clinically valuable predictive performance. From a theoretical perspective, extreme learning machine (ELM) randomly generates hidden layer weights and analytically solves the output weights, avoiding the complex gradient-based iterative process in traditional neural networks, which grants it faster training speed and stronger representation capacity. Building on this, the introduction of the Bayesian framework not only enables probabilistic modeling of model parameters and provides uncertainty estimation but also effectively suppresses overfitting through prior and posterior constraints. This combination allows Bayesian ELM to maintain efficient training while better balancing model complexity and generalization, thereby demonstrating stronger robustness and stability when applied to heterogeneous multi-center medical data.

Despite the promising results, our study has some limitations. First, the implementation of strict inclusion and exclusion criteria for samples could introduce bias in sample selection, potentially affecting model training. Second, the study includes only TBG, a specific type of benign nodule, and misses other benign nodules such as inflammatory pseudotumors, hamartomas, and fibromas. Third, the aggregation of local models in the Reptile stages dose not account for differences in data distribution across hospitals, potentially leading to suboptimal global models in the Reptile stage and affecting the performance of the robust local models trained in the next stage.

## Conclusion

The PFLS proposed in this study facilitates collaborative training across multiple hospitals while maintaining the data privacy of each hospital. It effectively mitigates the model overfitting caused by insufficient samples from a single hospital. Moreover, the personalizing process of local model address the heterogeneity of data across hospitals, which cannot be adequately performed by a single global model. The resulting robust local models show excellent discrimination between LAC and TBG, providing invaluable assistance to clinicians in improving diagnostic accuracy.

## Data Availability

Due to institutional policies and patient privacy regulations, the data are not publicly available. Access to the dataset is restricted and can only be granted upon reasonable request and with approval from the corresponding ethics committees of the participating institutions. Requests to access the datasets should be directed to XMC: 3897001254@qq.com.
