# Correction: A meta-learning-based robust federated learning for diagnosing lung adenocarcinoma and tuberculosis granulomas

**DOI:** 10.3389/fonc.2026.1806312

**Published:** 2026-02-18

**Authors:** Yuyao Chen, Lei Liu, Bao Feng, Yehang Chen, Jun Xu, Huan Lin, Kunwei Li, Xiaodong Chen, Yuting Ke, Haoyang Zhou, Qinghui Hu, Qinggeng Jin, Wansheng Long, Qiong Li, Xiangmeng Chen

**Affiliations:** 1 Department of Medical Imaging, Nanxishan Hospital of Guangxi Zhuang Autonomous Region, Guilin, China; 2 Laboratory of Artificial Intelligence of Biomedicine, Guilin University of Aerospace Technology, Guilin, China; 3 Department of Radiology, Guangdong Provincial People’s Hospital, Guangzhou, China; 4 Department of Radiology, Fifth Affiliated Hospital of Sun Yat-sen University, Zhuhai, China; 5 Department of Radiology, Affiliated Hospital of Guangdong Medical University, Zhanjiang, China; 6 School of Electrical Engineering, Guangxi University, Nanning, China; 7 Department of Radiology, Jiangmen Central Hospital, Jiangmen, China; 8 Department of Radiology, Sun Yat-sen University Cancer Center, Guangzhou, China

**Keywords:** lung adenocarcinoma, tuberculosis granuloma, solitary pulmonary solid nodules, SPSNs, meta-learning, federated learning, CT images, personalized federated learning signatures

Author “Xiangmeng Chen” was erroneously assigned to affiliation “Department of Medical Imaging, Nanxishan Hospital of Guangxi Zhuang Autonomous Region, Guilin, China”. This affiliation has now been removed for author “Xiangmeng Chen”.

Affiliation “Department of Radiology, Jiangmen Central Hospital, Jiangmen, China” was omitted from the paper. This affiliation has now been added for author “Xiangmeng Chen”.

Affiliation “1 Department of Medical Imaging, Nanxishan Hospital of Guangxi Zhuang Autonomous Region, Guilin, China” was erroneously given as “1 Nanxishan Hospital of Guangxi Zhuang Autonomous Region, Guilin, China”.

Affiliation “2 Laboratory of Artificial Intelligence of Biomedicine, Guilin University of Aerospace Technology, Guilin, China” was erroneously given as “2 Guilin University of Aerospace Technology, Guilin, China”.

Affiliation “3 Department of Radiology, Guangdong Provincial People’s Hospital, Guangzhou, China” was erroneously given as “3 Guangdong Provincial People’s Hospital, Guangzhou, China”.

Affiliation “4 Department of Radiology, Fifth Affiliated Hospital of Sun Yat-sen University, Zhuhai, China” was erroneously given as “4Department of Radiology, Fifth Affiliated Hospital Sun Yat-sen University, Zhuhai, China”.

Affiliation “5 Department of Radiology, Affiliated Hospital of Guangdong Medical University, Zhanjiang, China” was erroneously given as “5 Affiliated Hospital of Guangdong Medical University Department of Radiology, Zhanjiang, China”.

Affiliation “6 School of Electrical Engineering, Guangxi University, Nanning, China” was erroneously given as “6 Guangxi University School of Electrical Engineering, Nanning, China”.

Affiliation “7 Department of Radiology, Jiangmen Central Hospital, Jiangmen, China” was erroneously given as “7 Jiangmen Central Hospital, Jiangmen, China”.

Affiliation “8 Department of Radiology, Sun Yat-sen University Cancer Center, Guangzhou, China” was erroneously given as “8 Sun Yat-Sen University Cancer Center Department of Radiotherapy, Guangzhou, China”.

Authors Yuyao Chen and Lei Liu were erroneously omitted as equal contributing authors.

The original version of this article has been updated.

