# Supplementary material for: A meta-learning-based robust federated learning for diagnosing lung adenocarcinoma and tuberculosis granulomas
Source: Front Oncol. 2025 Sep 26;15:1666937. doi: 10.3389/fonc.2025.1666937 (PMC12510853; doi:10.3389/fonc.2025.1666937)
Supplement: Supplementary file 1 [file DataSheet1.docx]

## Supplementary S1: Sample Inclusion and Exclusion Criteria

The inclusion criteria were: (a) radical surgical resected SPSNs with final histopathological diagnosis confirmed LAC and LGN; (b) the diameter of the SPSNs ≤ 30 mm; (c) primary thoracic CT images with slice thickness 0.625-3.0 mm in the axial section; and (d) interval between preoperative thoracic CT examination and operation of less than 1 month.

The exclusion criteria for this study included: (a) calcified nodules or solid nodules with a satellited patchy opacity that represented chronic inflammatory disease; (b) sub solid nodules in the nodule attenuation subtype; (c) thoracic CT images with artifacts that did not meet the diagnostic requirements; and (d) patients with a malignant tumors history.

**Supplementary S2: CT Image Acquisition and Evaluation**

The chest CT images studied in this research were obtained using six devices: Somatom Definition Force (Siemens Medical Solutions; Germany), 64-detector-row CT scanner Aquilion One (Toshiba Medical Systems; Japan), GE Discovery (GE Healthcare; USA), dual-energy Somatom Flash (Siemens Medical Systems; Germany), PHILIPS Ingenuity (Philips Medical Systems; USA), and Lightspeed 16 (General Electric; USA). During the scan, patients were placed in the supine position and a craniocaudal scan of the entire chest was performed, covering the area from the thoracic inlet to the bilateral adrenal glands. CT images were taken during the breath-hold. The scanning procedure was performed in spiral mode with a collimation of 16 mm x 0.75 mm and a spacing from 0.875 to 1.5. The tube voltage was fixed at 120 kVp, and the mAs was automatically adjusted according to the patient's weight. Standard and high-resolution algorithms were employed for image reconstruction with a slice thickness of 1.0-3.0 mm and an interslice gap of 0.8-3.0 mm.

The CT images were independently assessed by two chest radiologists who were unaware of the additional clinical information of the patients. The CT images were evaluated using lung window settings (window width of 1500 HU and window level of -600 HU) and mediastinal window settings (window width of 300 HU and window level of 40 HU). During the evaluation, the radiologists focused on the following clinical factors and subjective CT findings: (a) nodule location (upper, middle or lower), (b) nodule size (measuring the longest diameters and its perpendicular diameters on the maximum cross-section, and calculating the average value), (c) shape of lesion margin, (d) lobulated shape, and (e) spiculated sign. Any discrepancies identified in the independent evaluations were addressed through discussion until a consensus was reached.

## Supplementary S3: Construction of federated learning based on model agnostic meta-learning

In the training process of the proposed method, each hospital is treated as a local client, and in addition to that, a global server responsible for aggregating all local models is required. Each client utilizes the ResNet18 as the local model. Since it is a binary classification problem to distinguish between tuberculosis and lung adenocarcinoma, the output dimension of the ResNet18 model should be set to 2. Each local client divides its local data into three sets: a training set, a validation set, and a test set, with a consistent ratio of both classes in each data set. The entire training process can be sequentially divided into three stages: FedAvg stage(federated average algorithm stage), Reptile stage (meta-learning-based federated learning stage), and the personalize stage(fine-tuning stage). In the FedAvg stage, the global server uses weighted averaging to aggregate the local models from each local client according to the Federated Averaging Algorithm (FedAvg). This aggregation process results in the initial global model for the next stage. Then, at the Reptile stage, the global server treats the local training process of the local clients as a task and aggregates the local models using the Reptile algorithm to compute a global model that can be easily fine-tuned by all the local clients. During the personalized stage, each local client fine-tunes the global model from the reptile stage using local data to acquire a personalized local model that is suitable for the local environment and has good generalization ability.

### FedAvg stage

The initial parameters of the local and global models are the same in the first iteration of the FedAvg stage and are trained on the ImageNet datasets. In the first step of an iteration, each local client trained its local model based on its own data sets using the stochastic gradient descent optimizer. In this step, the learning rate, batch size, and epochs of the optimizer are set to 0.0001, 32, and 60, respectively. After the training of all local clients, the gradients of the parameters of the local models are uploaded to the global client and weighted by the global client according to the following equation:

$$g_{FedAvg}=\frac{1}{n}\sum_{i=1}^{C} n_{i}g_{i,f} (1)$$

Here, *i* represents a local clients, $g_{i,f}=(W_{i,0}-W_{i,inner\_epoch})$ is the gradients of the *i*th local model in the FedAvg stage, $W_{i,inner\_epoch}$ is the parameters of the *i*th local model after the local training, $W_{i,0}$ is the parameters of the *i*th local model at the initial, $n_{i}$ is the number of samples of the *i*th client, *C* is the number of local clients, $n=\sum_{i=1}^{n_{p}} n_{i}$ is the total number of samples. Then, the global server updates the parameters of the global model $W_{gf}^{t+1}$ for next iteration as:

$$W_{gf}^{t+1}=W_{gf}^{t}-\eta\times g_{FedAvg} (2)$$

Here, $\eta$ is the hyperparameter, $t$ represents the current iteration. Subsequently, the global server distributes the parameters of the global model $W_{gf}^{t+1}$ to the local clients as the initial parameters of the local models $W_{i,0}$ in the next iteration. After several iterations, the last iteration parameters of the global model $W_{gf}$ is achieved and used as initial parameter of models in the next Reptile stage.

### Reptile stage

Compared to the FedAvg stage, the local clients of the Reptile stage utilize the Adam optimizer to update the parameters of the local models in the first step of each iteration. Also, the aggregation algorithm and updating process of the Reptile stage is different from the FedAvg stage and does not involve the number of samples. For aggregating the parameters of local models, the global client of the Reptile stage treats the local clients as different tasks of meta-learning and applies the Reptile algorithm as:

$$g_{Reptile}^{t}=\frac{1}{n_{p}}\sum_{i=1}^{n_{p}} g_{i,r}^{t} (3)$$

Here, $g_{i,r}^{t}$ is the gradient of the *i*th local model in the current iteration of Reptile stage. Then, the global server updates the parameters of the global model $W_{gr}^{t+1}$ for next iteration as with momentum as:

$$v_{t}=\gamma\times v_{t-1}+\eta\times g_{Reptile}^{t} (4)$$

$$W_{gr}^{t+1}=W_{gr}^{t}-v_{t} (5)$$

Here, $\eta$ and $\gamma$ are the hyperparameters, $t$ represents the current iteration. After the Reptile stage, the last parameters of global model are dispatched to each local client and fine-tuned by the local clients through its local data in the next personalized stage.

### Personalized stage

In the Personalize stage, the local clients do not share any data to the global server, and only fine-tune the local models with their own data sets based on the stochastic gradient descent algorithm. And the initial parameters of the local models are the final parameters of the global model of the Reptile stage.

During the whole training process, the raw data of a local client or hospital is never shared with the global server and other local clients, which ensures the security and privacy of the local data. The global server performs aggregation operations on the parameters of the local model so that the local clients can share the training results, effectively avoiding overfitting when the data samples of a single client are too small. The Reptile stage creates ideal conditions for rapid fine-tuning of the local model, and the Personalize stage can effectively solve the problem of data heterogeneity among different hospitals or centers.

### Experimental setup

FedAvg stage: using SGD with learning rate 0.0001, weight_decay 0.0001, loss function is CrossEntropyLoss, epochs and batch size of 32 as the client optimizer, and SGD with momentum of 1 and learning rate 1.0 as the server optimizer.

Reptile stage: using SGD with learning rate 0.0001, weight_decay 0.0001, loss function is CrossEntropyLoss, epochs 30, and batch size of 32.

Personalized stage: using SGD with learning rate 0.0001, weight_decay 0.0001, loss function is CrossEntropyLoss, epochs 30, and batch size of 32 as the client optimizer.

## Supplementary S4: Feature Extraction

After feeding a preprocessed 3-channel CT slice into the resnet18, the outputs of each convolutional layer are extracted, which are called the tensor in this work. There are a total of 17 convolutional layers in the resnet18, so 17 tensors can be obtained. Each of these 17 tensors is transformed into 17 vectors and these vectors are concatenated to finally obtain a feature vector of a CT slice, which has 3904 elements as shown in Figure 1 of the Supplementary Appendix. A lesion region has multiple CT slices, each of which can be input into the resnet18 and a feature vector is obtained by vectorization and concatenation operations. The feature vectors of all the CT slices of the lesion are averaged to obtain the feature vector of the lesion region and complete the process of feature extraction.


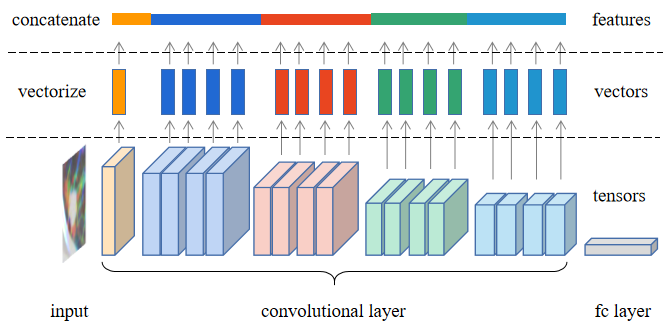


Figure S1 Feature extraction of an image from the resnet18

**Supplementary S5:The pseudocode of the PFLS Algorithm Framework**

| PFLS Algorithm Framework | | |
| --- | --- | --- |
| FedAvg Stage | Reptile Stage | Personalization Stage |
| Require: Local epochs E, learning rate β | Require: Local adaptation steps K, learning rate β | Require: Fine-tuning steps P, learning rate β |
| ClientUpdate(θ, Dᵢ, β) | InnerLoop(θ, Dᵢ, K, β) | Personalize(θ, Dᵢ, P, β) |
| Split dataset Dᵢ into batches | Initialize θᵢ ← θ | Initialize θᵢ ← θ |
| for each local epoch e = 1…E: | for step k = 1…K: | for step p = 1…P: |
| for each batch b ∈ Dᵢ: | Sample batch Dᵢ,ₖ from Dᵢ | Sample batch b ∈ Dᵢ |
| θᵢ ← θᵢ - β ∇L(θᵢ, b) | θᵢ ← θᵢ - β ∇L(θᵢ, Dᵢ,ₖ) | θᵢ ← θᵢ - β ∇L(θᵢ, b) |
| return gᵢ = θᵢ - θ | end for | end for |
| end function | return gᵢ = θᵢ - θ | return θᵢ |
| ServerUpdate: | ServerUpdate: | Evaluation: |
| θ ← θ + α (Σ wᵢ gᵢ / Σ wᵢ) | θ ← θ + (1/\|C\|) Σ gᵢ | Evaluate θᵢ on local test set |

**Supplementary S6: FedAvg model and ILM experimental setup**

FedAvg model: using SGD with learning rate 0.01, loss function is CrossEntropyLoss, epochs and batch size of 32 as the client optimizer, and SGD with momentum of 1 as the server optimizer.

ILM: using SGD with learning rate 0.000001, weight-decay 0.00000001, loss function is CrossEntropyLoss, batch size of 256 as the client optimizer, and SGD with momentum of 0.9.

**Table S1 The personalized federated learnng signature details of different cohorts**

|  | Total number of feature | Number of significant features by Mann-Whitney U test | Number of neurons of hidden layer of SB-ELM |
| --- | --- | --- | --- |
| Cohort 1 | 3904 | 1142 | 69 |
| Cohort 2 | 3904 | 438 | 33 |
| Cohort 3 | 3904 | 2641 | 20 |
| Cohort 4 | 3904 | 2934 | 28 |
| Cohort 5 | 3904 | 77 | 44 |

**Table S2 The NRI index of PFLS relative to FedAvg and ILM in the test sets**

|  | FedAvg | ILM | CM |
| --- | --- | --- | --- |
| PFLS of Cohort 1 | 0.664, *p* value = 0.002 | 0.716, *p* value < 0.001 | 0.478, *p* value = 0.024 |
| PFLS of Cohort 2 | 0.354, *p* value = 0.333 | 0.477, *p* value = 0.173 | 0.631, *p* value = 0.069 |
| PFLS of Cohort 3 | 1.263, *p* value < 0.001 | 0.969, *p* value < 0.001 | 1.204, *p* value < 0.001 |
| PFLS of Cohort 4 | 0.844, *p* value < 0.001 | 0.699, *p* value < 0.001 | 0.717, *p* value < 0.001 |
| PFLS of Cohort 5 | 0.287, *p* value = 0.388 | 0.324, *p* value = 0.315 | 1.154, *p* value < 0.001 |

**Table S3 The IDI index of PFLS relative to FedAvg and ILM in the test sets**

|  | FedAvg | ILM | CM |
| --- | --- | --- | --- |
| PFLS of Cohort 1 | 0.038, *p* value < 0.001 | 0.039, *p* value < 0.001 | 0.041, *p* value = 0.002 |
| PFLS of Cohort 2 | 0.016, *p* value = 0.503 | 0.022, *p* value = 0.205 | 0.048, *p* value = 0.009 |
| PFLS of Cohort 3 | 0.069, *p* value < 0.001 | 0.061, *p* value < 0.001 | 0.091, *p* value < 0.001 |
| PFLS of Cohort 4 | 0.056, *p* value < 0.001 | 0.045, *p* value < 0.001 | 0.060, *p* value < 0.001 |
| PFLS of Cohort 5 | 0.066, *p* value = 0.014 | 0.054, *p* value = 0.069 | 0.092, *p* value < 0.001 |

**Table S4 The FedAvg model details of different cohorts**

|  | Total number of feature | Number of significant features by Mann-Whitney U test | Number of neurons of hidden layer of SB-ELM |
| --- | --- | --- | --- |
| Cohort 1 | 3904 | 259 | 49 |
| Cohort 2 | 3904 | 28 | 9 |
| Cohort 3 | 3904 | 755 | 34 |
| Cohort 4 | 3904 | 3530 | 27 |
| Cohort 5 | 3904 | 17 | 6 |

**Table S5 The local deep learning model (ILM) details of different cohorts**

|  | Total number of feature | Number of significant features by Mann-Whitney U test | Number of neurons of hidden layer of SB-ELM |
| --- | --- | --- | --- |
| Cohort 1 | 3904 | 414 | 14 |
| Cohort 2 | 3904 | 867 | 9 |
| Cohort 3 | 3904 | 1748 | 19 |
| Cohort 4 | 3904 | 2001 | 37 |
| Cohort 5 | 3904 | 158 | 39 |

Table S6. Performance of the centralized MDCM

| Methods | Center | AUC  (95% CI) | Sensitivity | Specificity | Accuracy | PPV | NPV |
| --- | --- | --- | --- | --- | --- | --- | --- |
| Centralized | Train | 0.731  (0.686-0.776) | 0.853  (361/423) | 0.513  (96/187) | 0.749  (457/610) | 0.799  (361/452) | 0.608  (96/158) |
|  | Test | 0.704  (0.648-0.760) | 0.819  (235/287) | 0.442  (57/129) | 0.702  (292/416) | 0.765  (235/307) | 0.523  (57/109) |

Table S7. Performance of the centralized MDCM trained on merged data and validated on each site’s test set

| Center | Set | AUC  (95% CI) | Sensitivity | Specificity | Accuracy | PPV | NPV |
| --- | --- | --- | --- | --- | --- | --- | --- |
| Cohort 1 | Train | 0.784  (0.702-0.866) | 0.677  (86/127) | 0.794  (27/34) | 0.702  (113/161) | 0.925  (86/93) | 0.397  (27/68) |
|  | Test | 0.752  (0.630-0.857) | 0.588  (50/86) | 0.750  (18/23) | 0.624  (68/109) | 0.893  (50/56) | 0.340  (18/53) |
| Cohort 2 | Train | 0.855  (0.742-0.968) | 0.838  (31/37) | 0.786  (11/14) | 0.824  (42/51) | 0.912  (31/34) | 0.647  (11/17) |
|  | Test | 0.842  (0.698-0.987) | 0.885  (23/26) | 0.600  (6/10) | 0.806  (29/36) | 0.852  (23/27) | 0.667  (6/9) |
| Cohort 3 | Train | 0.847  (0.752-0.943) | 0.898  (44/49) | 0.714  (15/21) | 0.843  (59/70) | 0.880  (44/50) | 0.750  (15/20) |
|  | Test | 0.825  (0.701-0.950) | 0.824  (28/34) | 0.600  (9/15) | 0.755  (37/49) | 0.824  (28/34) | 0.600  (9/15) |
| Cohort 4 | Train | 0.785  (0.727-0.844) | 0.833  (155/186) | 0.635  (61/96) | 0.766  (216/282) | 0.816  (155/190) | 0.663  (61/92) |
|  | Test | 0.746  (0.673-0.818) | 0.768  (96/125) | 0.594  (38/64) | 0.709  (134/189) | 0.787  (96/122) | 0.567  (38/67) |
| Cohort 5 | Train | 0.854  (0.744-0.964) | 0.792  (19/24) | 0.773  (17/22) | 0.783  (36/46) | 0.792  (19/24) | 0.773  (17/22) |
|  | Test | 0.820  (0.661-0.979) | 0.412  (7/17) | 0.875  (14/16) | 0.636  (21/33) | 0.778  (7/9) | 0.583  (14/24) |

*
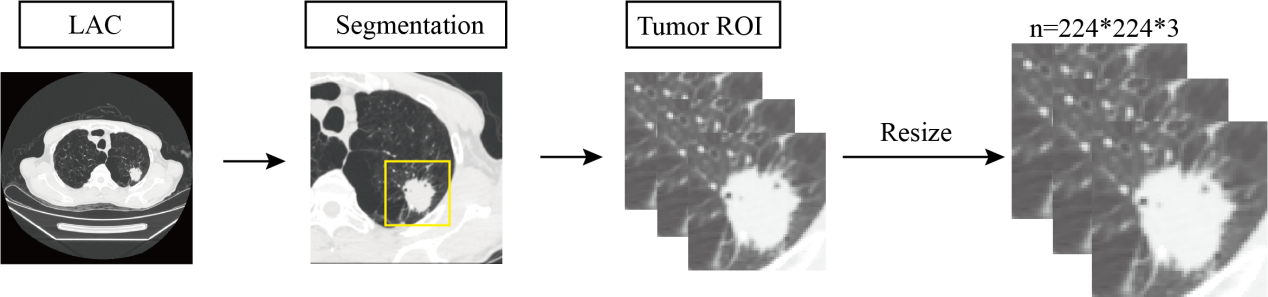
*

Figure S1. Data pre-processing

**
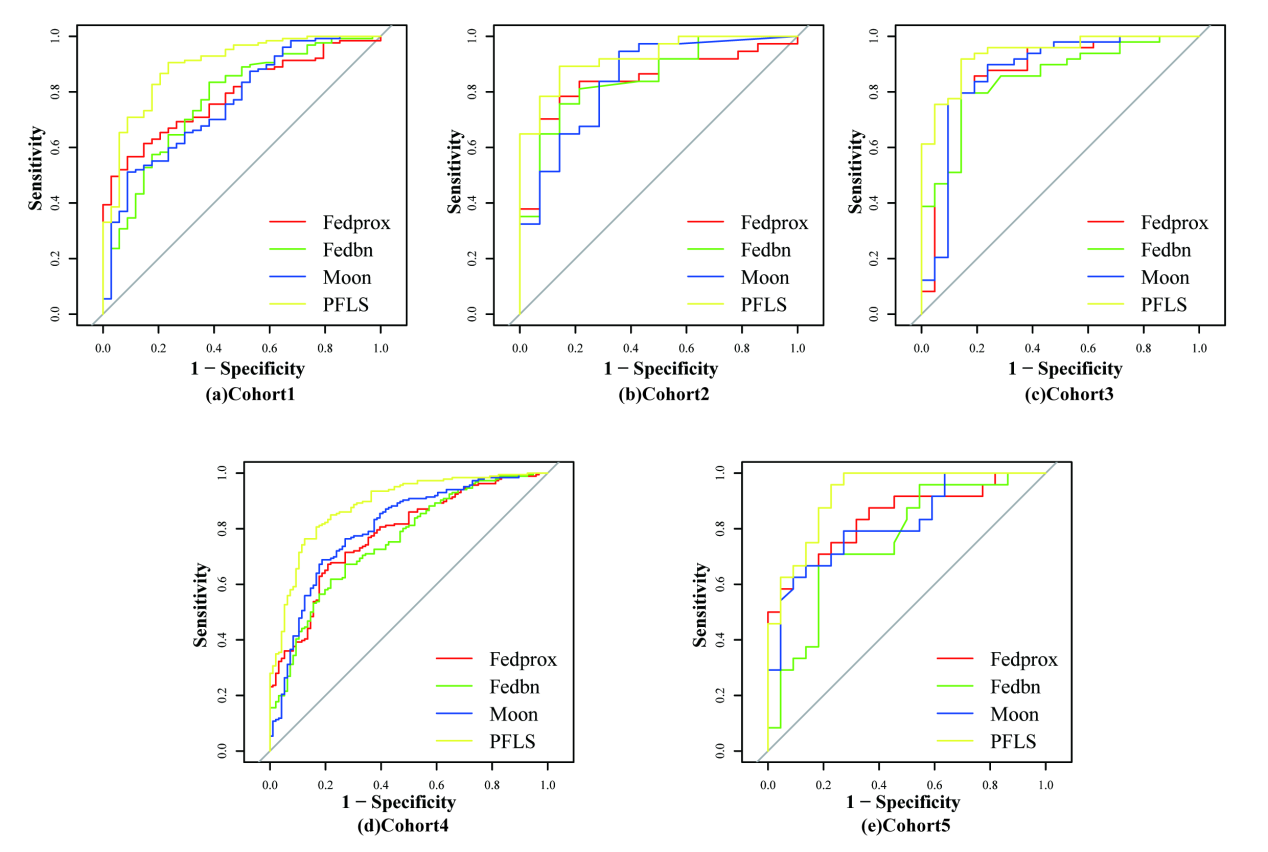
**

Figure S2. ROC of training set of five cohorts under different FL algorithms

*
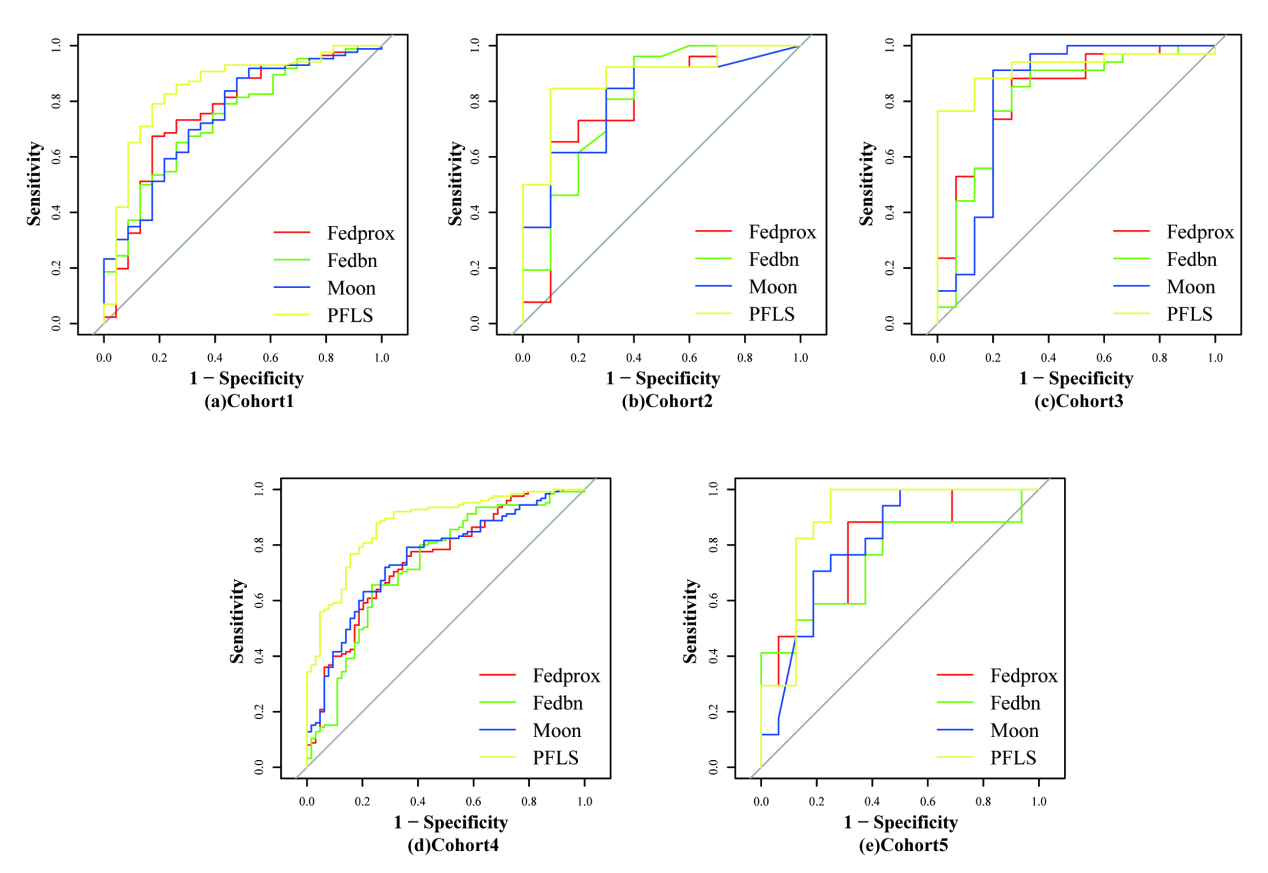
*

Figure S3. ROC of test set of five cohorts under different FL algorithms

*
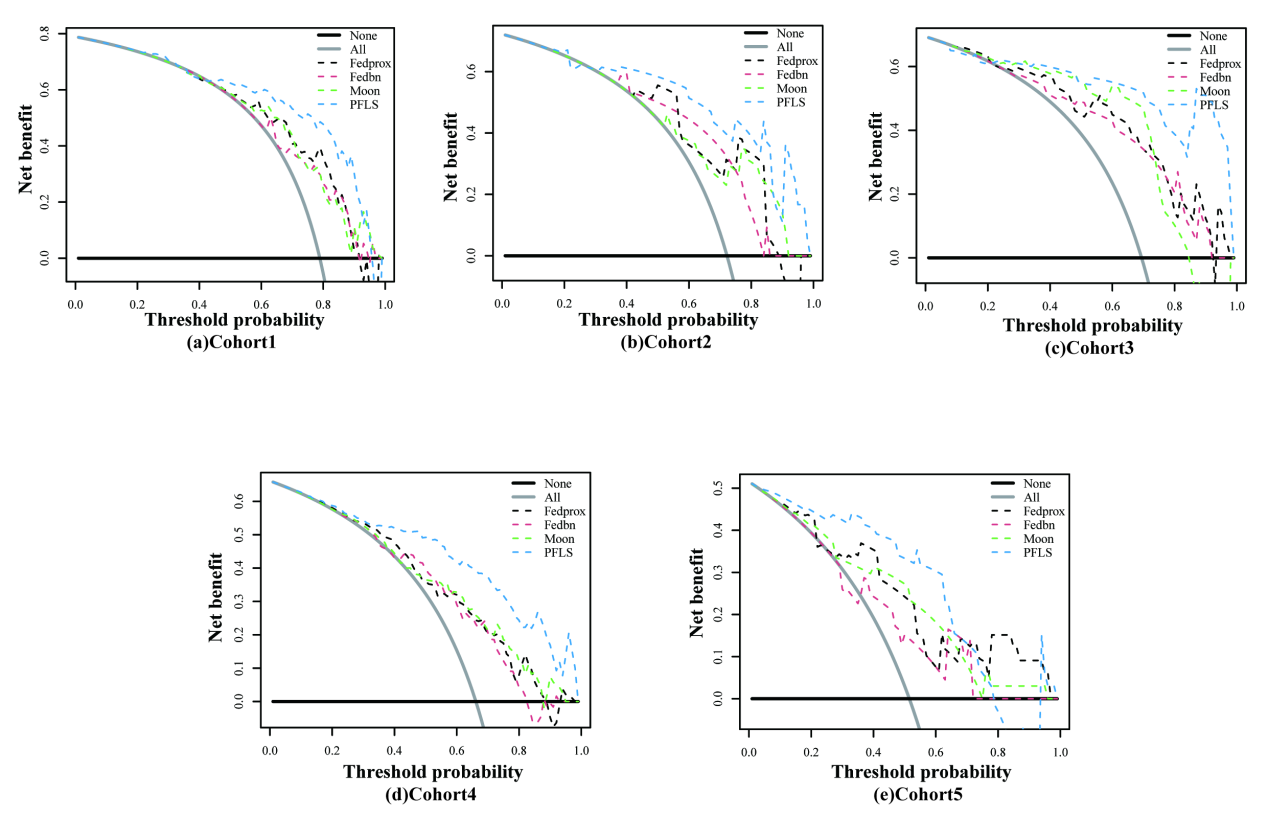
*

Figure S4. Decision curve analysis of test set of five cohorts under different FL algorithms
